# Draft Genome Sequence of an Oscillatorian Cyanobacterium, Strain ESFC-1

**DOI:** 10.1128/genomeA.00527-13

**Published:** 2013-08-01

**Authors:** R. Craig Everroad, Dagmar Woebken, Steven W. Singer, Luke C. Burow, Nikos Kyrpides, Tanja Woyke, Lynne Goodwin, Angela Detweiler, Leslie Prufert-Bebout, Jennifer Pett-Ridge

**Affiliations:** Exobiology Branch, NASA Ames Research Center, Moffett Field, California, USAa; Departments of Chemical Engineering and Civil and Environmental Engineering, Stanford University, Stanford, California, USAb; Earth Sciences Division, Lawrence Berkeley National Laboratory, Berkeley, California, USAc; DOE Joint Genome Institute, Walnut Creek, California, USAd; Los Alamos National Laboratory, Los Alamos, New Mexico, USAe; Physical and Life Sciences Directorate, Lawrence Livermore National Laboratory, Livermore, California, USAf

## Abstract

The nonheterocystous filamentous cyanobacterium strain ESFC-1 has recently been isolated from a marine microbial mat system, where it was identified as belonging to a recently discovered lineage of active nitrogen-fixing microorganisms. Here, we report the draft genome sequence of this isolate. The assembly consists of 3 scaffolds and contains 5,632,035 bp with a GC content of 46.5%.

## GENOME ANNOUNCEMENT

Nitrogen-fixing filamentous cyanobacteria are central components of intertidal microbial mat communities (1, 2). Recently, a new and diverse lineage of filamentous nitrogen-fixing cyanobacteria lacking both heterocysts and an extracellular sheath was identified as the major cyanobacterial diazotroph in the intertidal microbial mats at Elkhorn Slough, Moss Landing, California (3). An isolate of this lineage, strain ESFC-1, shares only a low 16S rRNA gene identity (<95%) with other identified cyanobacteria.

Strain ESFC-1 was isolated from the upper 2 mm of mat samples originating from Elkhorn Slough, California (36°48′46.61′′N, 121°47′4.89′′W). For isolation, mat material was plated onto nitrogen-free ASN and modified ASN agar plates and was subsequently rendered pure in liquid ASN (3, 4). High-molecular-weight genomic DNA was isolated through lysis with lysozyme, proteinase K, and SDS based on the protocol for bacterial genomic DNA isolation using cetyltrimethylammonium bromide (CTAB) provided by the Joint Genome Institute (JGI) (http://my.jgi.doe.gov/general/protocols/JGI-Bacterial-DNA-isolation-CTAB-Protocol-2012.pdf). RNA was digested with RNase according to this protocol, and 50 µg of DNA was provided for sequencing. The draft genome sequence of strain ESFC-1 was generated by the U.S. Department of Energy (DOE) JGI using Illumina sequencing technology (5). General aspects of library construction and sequencing performed by JGI are available at http://www.jgi.doe.gov/. Both an Illumina short-insert paired-end library with an average insert size of 222 bp, which generated 15,283,374 reads, and an Illumina long-insert paired-end library with an average insert size of 7,791 bp, which generated 18,062,354 reads, were constructed and sequenced, totaling 4,099 Mbp of Illumina data (6).

The initial draft data were assembled with Allpaths, version r38445 (7), and the consensus was computationally shredded into 10-kbp overlapping fake reads (shreds). The Illumina draft data were also assembled with Velvet, version 1.1.05 (8), and the consensus sequences were computationally shredded into 1.5-kbp overlapping fake reads (shreds). The draft data were assembled again with Velvet using the shreds from the first assembly to guide the next assembly. The consensus from the second Velvet assembly was shredded into 1.5-kbp overlapping fake reads. Fake reads from the Allpaths assembly, both Velvet assemblies, and a subset of the Illumina CLIP paired-end reads were assembled using parallel Phrap version 4.24 (High Performance Software, LLC). Possible misassemblies were checked and manually corrected in Consed (9, 10, 11). The final assembly is based on 4,099 Mbp of Illumina draft data, with an average of 719× coverage of the genome. ESFC-1 was resolved to 3 scaffolds consisting of 5,431,811, 135,349, and 64,875 bp (5,632,035 bp total). Average GC content was 46.51%.

Automated annotation was performed with both the RAST annotation server and the Integrated Microbial Genomes (IMG) system (12, 13). IMG identified 4,914 candidate protein-encoding genes, of which 71.27% had a predicted function. The ESFC-1 genome contains 72 tRNA genes and 2 rRNA operons. These rRNA operons have an average GC content of 57%. As predicted by RAST, these operons (1,451 and 1,452 bp) are 98.5% similar. The closest sequence match to either small-subunit (SSU) gene is from the marine unicellular *Aphanocapsa* sp. HBC6 at 93.6% similarity (accession EU249123 [14]).

### Nucleotide sequence accession number. 

This whole-genome shotgun project has been deposited at DDBJ/EMBL/GenBank under the accession number ARCP00000000. The version described in this paper is the first version.
